# Nonanatomical Reduction of Femoral Neck Fractures in Young Patients (≤65 Years Old) with Internal Fixation Using Three Parallel Cannulated Screws

**DOI:** 10.1155/2021/3069129

**Published:** 2021-01-04

**Authors:** Guanglei Zhao, Changquan Liu, Kangming Chen, Jinyang Lyu, Jie Chen, Jingsheng Shi, Feiyan Chen, Yibing Wei, Siqun Wang, Jun Xia, Gangyong Huang

**Affiliations:** Department of Orthopedics, Huashan Hospital, Fudan University, Shanghai 200040, China

## Abstract

**Purpose:**

The study is aimed at investigating the association between different reduction classifications (anatomic reduction, positive buttress position reduction, and negative buttress position reduction) and two end points (complications and reoperations).

**Methods:**

The study retrospectively analyzed 110 patients undergoing internal fixation with three parallel cannulated screws from January 2012 to January 2019 in Huashan Hospital. Based on the principles of the “Gotfried reduction,” all enrolled patients were divided into three groups: anatomic reduction, positive buttress position reduction, and negative buttress position reduction intraoperatively or immediately after surgery. Clinical characteristics including age, sex, side, Garden classification, Pauwels classification, fracture level, reduction classification, Garden alignment index angles, cortical thickness index (CTI), tip-caput distance (TCD), angle of the inferior screw, and the two ending points (complications and reoperations) were included in the statistical analysis. The Mann-Whitney *U*-test, the chi-square test, Fisher's exact test, and multiple logistic regression analysis were used in the study.

**Results:**

Of the 110 patients included in our study, the mean ± standard deviation (SD) of age was 51.4 ± 10.4 years; 41 patients showed anatomic reduction, 35 patients showed positive buttress position reduction, and 34 patients showed negative buttress position reduction. For the outcomes, 24 patients (anatomic reduction: 6 [14.6%]; positive buttress position reduction: 5 [14.3%]; negative buttress position reduction: 13 [38.2%]) had complications, while 18 patients (anatomic reduction: 5 [12.2%]; positive buttress position reduction: 3 [8.6%]; negative buttress position reduction: 10 [29.4%]) underwent reoperations after surgery. In the multivariate logistic regression analysis of complications, negative buttress position reduction (negative buttress position reduction vs. anatomic reduction, OR = 4.309, 95%CI = 1.137 to 16.322, and *p* = 0.032) was found to be correlated with higher risk of complications. The same variable (negative buttress position reduction vs. anatomic reduction, OR = 5.744, 95%CI = 1.177 to 28.042, and *p* = 0.031) was also identified as risk factor in the multivariate logistic regression analysis of reoperations. However, no significant difference between positive reduction and anatomical reduction was investigated in the analysis of risk factors for complications, not reoperations.

**Conclusion:**

Positive buttress position reduction of femoral neck fractures in young patients showed a similar incidence of complications and reoperations compared with those of anatomic reduction. For irreversible femoral neck fractures, if positive buttress position reduction has been achieved intraoperatively, it is not necessary to pursue anatomical reduction; however, negative reduction needs to be avoided.

## 1. Introduction

Worldwide, 4.5 million patients suffer from hip fractures every year, and femoral neck fractures account for a large proportion of hip fractures [1]. For old patients (age > 65 years) with femoral neck fractures, hemiarthroplasty and total hip arthroplasty are the common treatment methods for patients with poor physical condition, while reduction and internal fixation can also be an effective therapy in patients with good physical condition [1, 2]. For young patients (age ≤ 65 years) with femoral neck fractures, reduction and internal fixation are always the first choice in order to keep the native hip joint [1, 3]. However, the risk of postoperative complications in young patients is high, especially the risk of fracture nonunion (9%), femoral head necrosis (14%), and femoral neck shortening (13-32%) [4–6]. Therefore, the issue of how to improve the prognosis of young patients with femoral neck fractures is still a challenge for orthopedists.

It is well-known that anatomical reduction is important for fracture patients, while sometimes it is difficult to achieve anatomical reduction for young patients with displaced femoral neck fractures [7, 8]. Performing multiple reductions or open reductions in pursuit of anatomical reduction may damage the blood supply of the site and then increase the risk of postoperative complications [7, 9]. In 2013, Gotfried et al. [10] proposed a nonanatomical reduction method called the “Gotfried reduction.” It is worth mentioning that good results were shown in a series of 5 patients (53-75 years old) treated by the “Gotfried positive buttress position reduction” method with a minimum 1-year follow-up (1-2 years). Recently, in a cohort of 46 patients (19-60 years old) with a mean follow-up of 22 months, Xiong et al. [8] also reported satisfactory results in patients with the “Gotfried positive buttress position reduction.” However, the number of cases in both studies was small, and there was no other relevant large sample size in the literature.

In this retrospective study, our purposes were to test the association between reduction classification (anatomic reduction, positive buttress position reduction, and negative buttress position reduction), other clinical variables, and two outcomes (complications and reoperations) in patients following closed reduction and internal fixation with three parallel cannulated screws.

## 2. Methods

### 2.1. Patient Selection

This study retrospectively analyzed patients in the database of the Orthopedics Department, Huashan Hospital, Fudan University. Inclusion criteria are as follows: (1) diagnosed with femoral neck fracture from January 2012 to January 2019, (2) age ≤ 65 years old (young patients), (3) undergoing closed reduction and internal fixation surgery (three parallel cannulated screws with inverted triangle distribution), (4) the reduction was of high quality (Garden alignment index was within the range of 155°-180° in both anteroposterior (AP) and lateral radiographs) [11], and (5) no decreased mobility and other severe hip diseases before femoral neck fracture. Exclusion criteria are as follows: (1) patients with pathological fractures, (2) follow-up less than 1 year, (3) patients with cognitive dysfunction or mental disorders, (4) patients with acetabular fractures, or (5) patients without complete data of radiographs. Finally, we included a total of 110 patients in our study. The study was approved by the Ethics Committee of Huashan Hospital, Fudan University (KY2019838).

### 2.2. Patient Management (Intraoperative and Postoperative)

The patients were placed on a traction bed in the supine position. Closed reduction was performed under fluoroscopy. After the confirmation of good reduction quality (evaluated by the Garden alignment index), three parallel cannulated screws were inserted for internal fixation [11]. Then, fluoroscopy was used again to make sure the reduction was of high quality (Garden alignment index was within the range of 155°-180° in both AP and lateral radiographs) and the screws were in good position (three cannulated screws were placed parallel to one another and perpendicular to the fracture line with inverted triangle distribution) [11].

The patients stayed in bed after the surgery and started rehabilitation exercise on the first day postoperatively. Weight-bearing walking started after the healing of fractures were shown on the X-rays during follow-up (from 6 weeks at the earliest to 3 months after surgery). The exercise was partially weight-bearing in the early stage, and then gradually changed to full weight-bearing.

### 2.3. Radiographic Assessment

All the patients had standard AP radiographs (supine position with the leg rotated 15° to 20° medially) of the hip preoperatively, AP and lateral fluoroscopies (the X-ray is perpendicular to the axis of the femoral neck) of the hip intraoperatively, and standard AP radiographs of the hip postoperatively. The radiographs were analyzed and measured through the hospital's imaging system (GE Medical Systems) by two experienced orthopedists. The two orthopedists were not involved in the surgery process.

**Figure 1:**
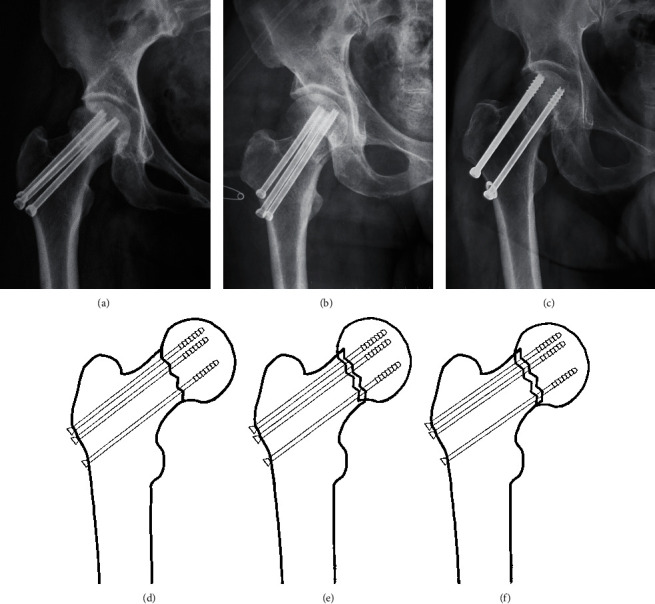
A pattern diagram showing (a, d) anatomic reduction, (b, e) positive buttress position reduction, and (c, f) negative buttress position reduction on AP radiograph. In positive buttress position reduction, the distal fracture segment is located inferiorly-medially to the lower-lateral part of the proximal fracture segment on AP radiographs. In negative buttress position reduction, the distal fracture segment was located superiorly-medially to lower-lateral part of the proximal fracture segment on AP radiographs.

**Figure 2:**
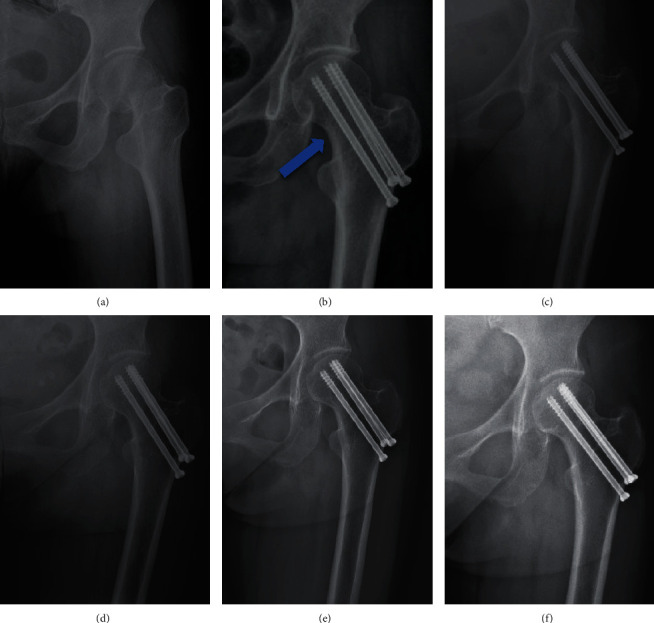
Follow-up of a female patient (49 years old) with anatomic reduction (a–f). (a) Presurgery. (b) Immediately after surgery: anatomic reduction (blue arrow). (c–f) 6 months, 9 months, 2 years, and 5 years after surgery: no complication occurred.

**Figure 3:**
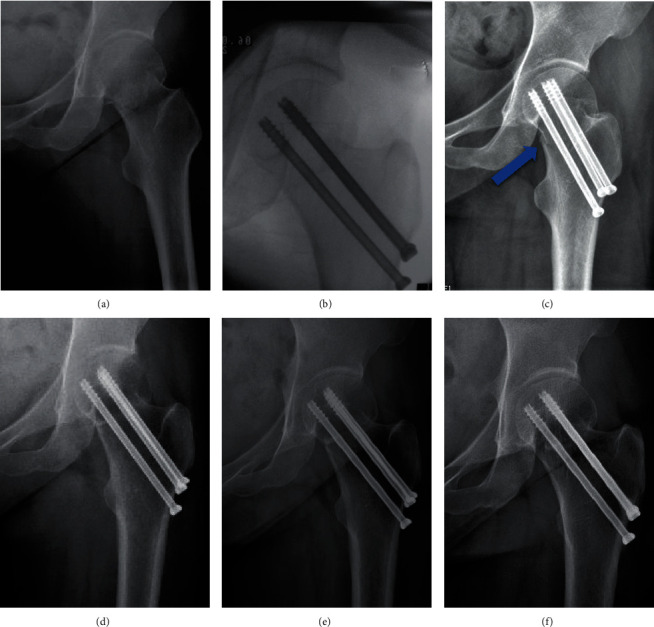
Follow-up of a female patient (36 years old) with positive buttress position reduction (a–f). (a) Presurgery. (b, c) Intraoperatively and immediately after surgery: positive buttress position reduction (blue arrow). (d–f) 6 months, 1 year, and 5 years after surgery: no complication occurred.

**Figure 4:**
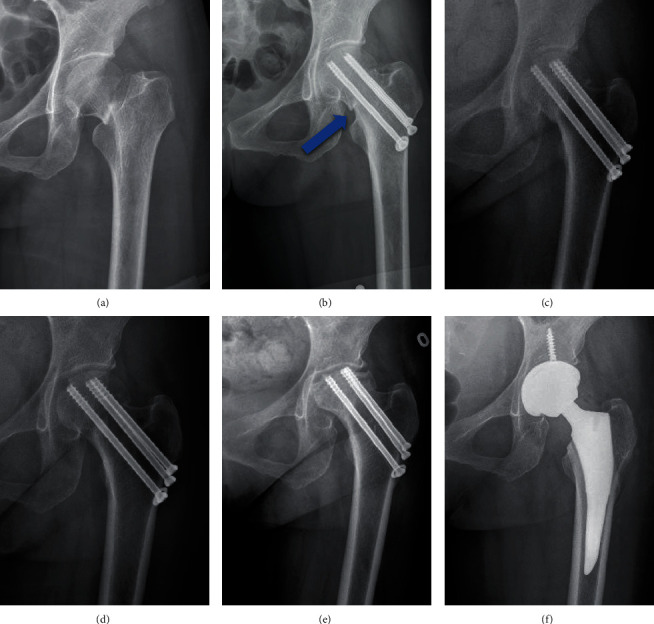
Follow-up of a female patient (51 years old) with negative buttress position reduction (a–f). (a) Presurgery. (b) Immediately after surgery: negative buttress position reduction (blue arrow). (c) 6 months after surgery: nonunion remained and perforation occurred. (d) 15 months after surgery: fracture healed, while perforation remained. (e) 2 years after surgery: avascular necrosis of the femoral head (AVN) occurred. (f) 2 years after surgery: total hip arthroplasty (THA) was performed.

**Figure 5:**
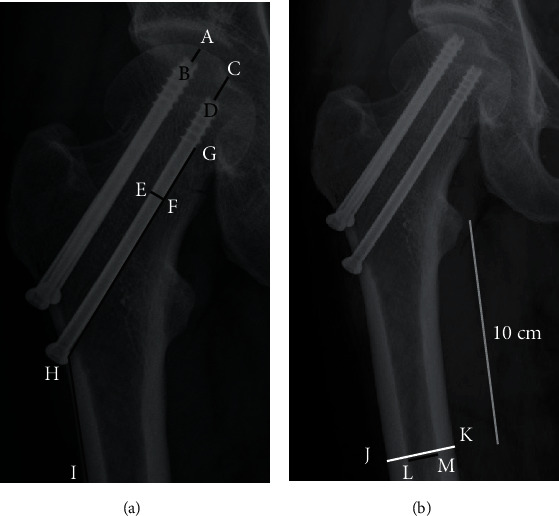
A pattern diagram of different radiographic measurements (a, b). (a) A-B and C-D: the tip-cartilage distance of each screw; E-F: the diameter of screw, used for the calibration of measurements; G-H-I: angle of inferior screw, the angle between the lateral cortex of the femoral shaft and the inferior screw. (b) Cortical thickness index (CTI) = (distance (J − K)–distance (L − M))/distance (J − K). CTI is defined as the ratio of the thickness of the cortical bone to the diameter of the femoral shaft 10 cm below the tip of trochanter minor.

The Garden classification, Pauwels classification, and fracture level were assessed using standard AP radiographs of the hip preoperatively [12, 13]. Based on the principles of the “Gotfried reduction” described previously, all the enrolled patients were divided into three groups: anatomic reduction, positive buttress position reduction (the distal fracture segment located inferiorly-medially to the lower-lateral part of the proximal fracture segment on AP radiographs), and negative buttress position reduction (the distal fracture segment located superiorly-medially to lower-lateral part of the proximal fracture segment on AP radiographs) (Figures 1–4) [8, 10]. The Garden alignment index was measured on the AP and lateral fluoroscopies intraoperatively to assess the quality of the reduction. Tip-caput distance (TCD) was defined as the smallest value of the tip-cartilage distance of the three screws (Figure 5(a)). The angle of the inferior screw was measured as the angle between the lateral cortex of the femoral shaft and the inferior screw (Figure 5(a)). Cortical thickness index (CTI) was defined as the ratio of the thickness of the cortical bone to the diameter of the femoral shaft 10 cm below the tip of the trochanter minor. CTI = (distance (J − K)–distance (L − M))/distance (J − K) (Figure 5(b)) [14].

### 2.4. Complications

#### 2.4.1. Displacement to Varus (>10°)

The femoral neck-shaft angle changing more than 10° postoperatively was defined as displacement to varus.

#### 2.4.2. Shortening (>5 mm)

The length of the femoral neck was measured on the basis of the method proposed by Zlowodzki et al. [15], and length change > 5 mm was regarded as a shortening of the femoral neck [8, 16].

#### 2.4.3. Avascular Necrosis (AVN) of Femoral Head

A Steinberg stage 2 or more on the radiographs after the operation was considered AVN in our study [17, 18].

#### 2.4.4. Nonunion

Fracture nonunion was defined as the presence of a fracture line on radiographs one year after surgery [17].

### 2.5. Reoperations

The simple removal of internal fixation implants was not considered as a reoperation. Reoperation was defined as conversion of internal fixation to total hip replacement, because only this type of reoperation was observed in our study.

### 2.6. Interobserver Reliability

Interobserver reliability for continuous variables (CTI, TCD, and the angle of the inferior screw) was measured using the intraclass correlation coefficient (ICC); interobserver reliability for categorical variables (reduction classification) was measured using the *κ* coefficient.

### 2.7. Statistical Analysis

Continuous variables were presented as the means ± standard deviations (SDs), while categorical variables were presented as frequencies with percentages (%).

The Mann-Whitney *U*-test (for continuous variables) and the chi-square test or Fisher's exact test (for categorical variables) were used to compare the clinical characteristics between the complication group and noncomplication group. Variables (*p* < 0.10 in univariate analyses) were further analyzed in multiple logistic regression analysis with complications as the dependent variable. The odds ratios (ORs) of the variables were shown with 95% confidence intervals (CIs). The same analysis method as above was used between the reoperation group and the nonreoperation group.

All analyses were performed using SPSS 24.0 by the author C.Q. Liu, and *p* < 0.05 (two-tailed) was regarded statistically significant.

## 3. Results

**Figure 6:**
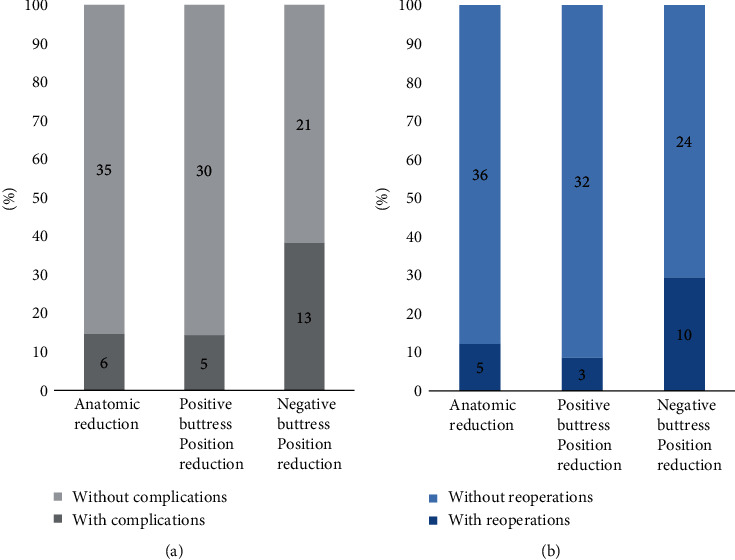
Distribution of (a) complications and (b) reoperations in different reduction methods. (a) The percentage of complications in anatomic reduction, positive buttress position reduction, and negative buttress position reduction was 14.6%, 14.3%, and 38.2%. (b) The percentage of reoperations in the three reduction methods was 12.2%, 8.6%, and 29.4%.

A total of 110 cases were included in the study. The mean ± SDs of age and follow-up were 51.4 ± 10.4 years and 27.1 ± 17.6 months, respectively. Of all the patients, 62 patients were male, while only 48 patients were female; 55 patients had the surgery on the right hip and the others (55 patients) on the left hip. The means (±SD) of the Garden alignment index angles were 164.3 ± 3.6° and 177.4 ± 2.3° on the AP and lateral X-rays, respectively. For the reduction classification, 41 patients showed anatomic reduction, 35 patients showed positive buttress position reduction, and 34 patients showed negative buttress position reduction (Table 1). Other radiographic variables, including the Garden classification, Pauwels classification, fracture level, CTI, TCD, and angle of the inferior screw, are also presented in Table 1. The interobserver reliability of the radiographic variables (reduction classification, CTI, TCD, and angle of the inferior screw) is shown in Table 2. A total of 24 patients (21.8%) had complications during follow-up, of whom 6 patients (14.6%) were anatomic reduction, 5 patients (14.3%) were positive buttress position reduction, and 13 patients (38.2%) were negative buttress position reduction. For reoperations, there were 18 patients undergoing total hip arthroplasty (THA) after surgery, of whom 5 patients (12.2%) were anatomic reduction, 3 patients (8.6%) were positive buttress position reduction, and 10 patients (29.4%) were negative buttress position reduction (Table 3 and Figure 6).

### 3.1. Univariate Analysis

In univariate analysis of complications, no significant differences were investigated in age, sex, side, Pauwels classification, fracture level, Garden alignment index angles, and TCD, while the Garden classification (*p* = 0.046), reduction classification (*p* = 0.020), CTI (*p* = 0.026), and angle of the inferior screw (*p* = 0.005) showed significant differences between the complication group and noncomplication group (Table 4).

In univariate analysis of reoperations, significant differences were shown in reduction classification (*p* = 0.020), CTI (*p* = 0.026), and angle of inferior screw (*p* = 0.005). For other variables (age, sex, side, Garden classification, Pauwels classification, fracture level, Garden alignment index angles, and TCD), no significant difference was found between the reoperation group and nonreoperation group, while the *p* value was 0.067 (*p* < 0.1) in the Garden classification (Table 4).

### 3.2. Multivariate Analysis

Variables (*p* < 0.1 in univariate analysis), including the Garden classification, reduction classification, CTI, and angle of the inferior screw, were selected for multivariate logistic regression analysis of complications and reoperations.

The multivariate logistic regression analysis revealed that three factors, including reduction classification (negative buttress position reduction vs. anatomic reduction, OR = 4.309, 95%CI = 1.137 to 16.322, and *p* = 0.032), CTI (0.5-0.6 vs. 0.4-0.5, OR = 0.048, 95%CI = 0.005 to 0.510, and *p* = 0.012), and angle of the inferior screw (> 125°, OR = 0.043, 95%CI = 0.004 to 0.498, and *p* = 0.012), were identified as independent prognostic factors for complications (Table 5).

These variables, including reduction classification (negative buttress position reduction vs. anatomic reduction, OR = 5.744, 95%CI = 1.177 to 28.042, and *p* = 0.031), CTI (0.5-0.6 vs. 0.4-0.5, OR = 0.033, 95%CI = 0.002 to 0.543, and *p* = 0.017), and angle of the inferior screw (> 125°, OR = 0.063, 95%CI = 0.006 to 0.659, and *p* = 0.021), were also significant in the multivariate logistic regression analysis of reoperations (Table 6).

## 4. Discussion

It was worth mentioning that, in our study, we investigated patients with positive reduction (14.3%) who had a similar proportion of complications compared to those patients with anatomical reduction (14.6%), and the former (8.6%) had an even lower proportion of reoperations compared to the latter (12.2%) (Table 3 and Figure 6). However, negative buttress position reduction showed worse results both in risk of complications (Table 5) and reoperations (Table 6) when compared to anatomic reduction.

The “Gotfried reduction” is a nonanatomical reduction method proposed by Gotfried et al. [10] in 2013, in which positive buttress position reduction is defined as the distal fracture segment located inferiorly-medially to the lower-lateral part of the proximal fracture segment on AP radiographs. The study by Gotfried et al. [10] included 5 patients (53 to 75 years) with the “Gotfried positive buttress position reduction” in the study and showed that these patients all had satisfactory results (no fracture redisplacement, nonunion, or AVN) in a minimum 1-year follow-up (1 to 2 years). Recently, another study by Xiong et al. [8] reported similar results (no significant difference existed in the complication rate between the “Gotfried positive buttress position reduction” and “anatomical reduction” groups) in a cohort of 46 patients (19-60 years) with short follow-up time (mean 22 months). Both studies had roughly verified the effectiveness of the “Gotfried positive buttress position reduction” with a small number of cases and short follow-up. In our study, we included the largest number of cases (a total of 110 patients) with the longest-term follow-up (mean 27.1 ± 17.6 months; range 1 to 5 years). All the patients had a high quality reduction (Garden alignment index was within the range of 155°-180° in both AP and lateral radiographs), and the means (±SD) of Garden alignment index angles were 164.3 ± 3.6° and 177.4 ± 2.3° on the AP and lateral X-rays (Table 1) [11]. It was worth mentioning that, other than the simple positive buttress position reduction group [10] or the positive buttress position reduction and anatomic reduction groups [8], the patients in our study were divided into three groups: anatomic reduction, positive buttress position reduction, and negative buttress position reduction according to the principles of the “Gotfried reduction.” In the results, we found that patients with positive buttress position reduction had a similar or even lower proportion of complications and reoperations as patients with anatomical reduction (Table 3), which was similar to the results of previous studies [8, 10]. In addition, we also found that patients with negative buttress position reduction were prone to a higher risk of complications (Table 5) and reoperations (Table 6) compared with those in anatomical reduction. These results showed that anatomic reduction as well as positive buttress position reduction was both acceptable, while negative buttress position reduction should be avoided, when performing internal fixation surgery in young patients with femoral neck fractures.

For unstable femoral neck fractures in young patients, many scholars have put forward a fixation method with three parallel cannulated screws and medial support plate, which showed that patients with this kind of fixation had a better prognosis than patients with simple three parallel cannulated screws [7, 17, 19–21]. Positive buttress position reduction, of which the proximal fracture segment is supported by the inferior femoral neck cortex, is similar to the fixation method listed above, while the former had less traumatic damage than the latter. It was worth mentioning that patients with positive buttress position reduction also showed good prognosis in our study. Moreover, Chang et al. [22] proposed the new theory of positive and negative buttress position reduction in patients with intertrochanteric fractures in 2015. A series of studies about the theory were subsequently published, which showed that the positive buttress position reduction had a better prognosis than the negative buttress position reduction [22–24]. These examples all had a certain supportive effect on the “Gotfried positive buttress position reduction” theory applied to the femoral neck fractures, and we did find that patients with positive buttress position reduction had similar results compared to patients with anatomic reduction, while patients with negative buttress position reduction showed worse results in contrast to positive buttress position reduction and anatomic reduction in our study.

It is worth mentioning that femoral neck stress fractures can sometimes occur in young patients, commonly seen in long-distance runners and military recruits [25]. Similarly, the presence of bone resorption and shear forces around the fracture line often lead to postoperative complications (displacement to varus, femoral neck shortening, etc.) during the healing of femoral neck fractures. Shear force around the fracture line can be reduced by weight loss, but bone resorption around the fracture line is often inevitable [8, 10]. This could explain that each type of reduction patients had some postoperative complications and reoperations in our study (Table 3). For positive buttress position reduction, the proximal fracture segment is supported by the inferior femoral neck cortex. This structure can reduce the effect of the shear force at the fracture line, convert the shear force into a force that promotes the healing of the lower part of the fracture line, and reduce the force at the bone-nail contact [26]. However, such structure exists neither in negative buttress position reduction patients nor in anatomical reduction patients. This was consistent with our results that the positive buttress position reduction group had only 5 (14.3%) complications and 3 (8.6%) reoperations, while the negative buttress position reduction and anatomical reduction groups had 13 (38.2%) complications and 10 (29.4%) reoperations and 6 (14.6%) complications and 5 (12.2%) reoperations, respectively (Table 3).

Recently, there have also been studies verifying the effectiveness of positive buttress position reduction through biomechanics and animal experiments [27, 28]. In 2019, Wang et al. [28] investigated that better biomechanical stability was shown in the positive buttress position reduction group compared to that in the negative buttress position reduction group in a finite element analysis. It was worth mentioning that they further quantified the range of positive buttress and concluded that the range of positive buttress should be controlled within 3 mm as much as possible. Furthermore, the same group proposed that positive buttress position reduction repaired the fracture site by promoting bone formation and blood vessel formation through the animal experiments of rabbits in 2020 [27].

In our research, we used the new indicator CTI, which has a strong correlation with bone mineral density (BMD) and is easier to obtain than the dual energy X-ray absorptiometry (DEXA) scan (gold standard) [14, 29]. We investigated that lower CTI was related to higher risk of complications (0.5-0.6 vs. 0.4-0.5, OR = 0.048, 95%CI = 0.005 to 0.510, and *p* = 0.012) (Table 5), as well as reoperations (0.5-0.6 vs. 0.4-0.5, OR = 0.033, 95%CI = 0.002 to 0.543, and *p* = 0.017) (Table 6), in our study. It was worth mentioning that a previous study by Nyholm et al. [30] also reported similar results in a cohort of 654 patients from the Danish Fracture Database. These results suggested that CTI might be used as a predictor of postoperative complications and reoperations in patients (≤65 years old) following internal fixation with three parallel cannulated screws. In our study, we also found that a higher angle of the inferior screw was a protective factor for postoperative complications (> 125°, OR = 0.043, 95%CI = 0.004 to 0.498, and *p* = 0.012) (Table 5) and reoperations (> 125°, OR = 0.063, 95%CI = 0.006 to 0.659, and *p* = 0.021) (Table 6), which was consistent with previous studies by Viberg et al. [31] in 2016 and Nyholm et al. [30] in 2020. A “too flat” angle of screw should be avoided in the process of surgery.

## 5. Limitations

Our research had its limitations. First, our research was a retrospective study in one orthopedic center, and we included a relatively small number of patients based on the inclusion and exclusion criteria. A prospective, multicenter study with more cases in the future could make the results more credible. Second, the positive buttress position reduction in this study was not quantified, and we do not know the optimal length of buttress cortex coverage. However, excessive coverage may lead to undesirable results. Studies designed to quantify the length of buttress cortex coverage can obtain more precise conclusions and better guide clinical practice. Third, no biomechanical or animal experiments were explored in our center before this study. Subsequent biomechanical or animal experiments are required to further verify the effectiveness of positive buttress and quantify the length of buttress cortex coverage. Last, we included patients with basicervical fractures in our study. As the biomechanics of this fracture are more similar with the intertrochanteric fracture rather than the femoral neck fracture, including such patients in the analysis might lead to misleading results. Researches excluding patients with basicervical fractures in the future are needed to eliminate this bias. For future directions, clinical research—large sample size and multicenter research can be carried out to verify the method; basic research—anatomy and mechanics studies can be used to improve the theoretical system, especially the length of buttress cortex coverage.

## 6. Conclusion

Positive buttress position reduction of femoral neck fractures in young patients showed a similar incidence of complications and reoperations compared with anatomic reduction. However, negative buttress position reduction presented worse results compared with anatomic reduction. For irreversible femoral neck fractures, if positive buttress position reduction has been achieved intraoperatively, it is not necessary to pursue anatomical reduction; however, negative reduction needs to be avoided.

## Figures and Tables

**Table 1:** Basic characteristics.

Variables	Total (*n* = 110)
Age (years)	51.4 ± 10.4
Sex	
Female	48 (43.6%)
Male	62 (56.4%)
Side	
Right	55 (50.0%)
Left	55 (50.0%)
Garden classification	
I, II	26 (23.6%)
III, IV	84 (76.4%)
Pauwels classification	
I	25 (22.7%)
II	55 (50.0%)
III	30 (27.3%)
Fracture level	
Subcapital	63 (57.2%)
Midcapital	39 (35.5%)
Basicervical	8 (7.3%)
Reduction classification	
Anatomic reduction	41 (37.3%)
Positive buttress position reduction	35 (31.8%)
Negative buttress position reduction	34 (30.9%)
Garden alignment index angles (°)	
AP	164.3 ± 3.6
Lateral	177.4 ± 2.3
CTI (every 0.1)	
<0.4	0
0.4-0.5	23 (20.9%)
0.5-0.6	60 (54.5%)
0.6-0.7	27 (24.6%)
>0.7	0
TCD	
<5 mm	82 (74.5%)
≥5 mm	28 (25.5%)
Angle of inferior screw	
<125°	7 (6.4%)
≥125°	103 (93.6%)
Follow-up (months)	27.1 ± 17.6

AP: anterior-posterior; CTI, cortical thickness index; TCD: tip-caput distance.

**Table 2:** Interobserver reliability.

Variables	ICC or *κ*	95% CI	*p*
Reduction classification, *κ*	0.904	0.835 to 0.973	<0.001
CTI, ICC	0.909	0.802 to 0.951	<0.001
TCD, ICC	0.822	0.741 to 0.878	<0.001
Angle of inferior screw, ICC	0.852	0.783 to 0.898	<0.001

CI: confidence interval; CTI: cortical thickness index; ICC: intraclass correlation coefficient; *κ*: kappa coefficient; TCD: tip-caput distance.

**Table 3:** Complications and reoperations.

Variables	Total (*n* = 110)	Anatomic reduction (*n* = 41)	Positive buttress position reduction (*n* = 35)	Negative buttress position reduction (*n* = 34)
Complications	24 (21.8%)	6 (14.6%)	5 (14.3%)	13 (38.2%)
Displacement to varus (>10°)	8 (7.3%)	2 (4.9%)	2 (5.7%)	4 (11.8%)
Shortening (>5 mm)	20 (18.2%)	5 (12.2%)	4 (11.4%)	11 (32.4%)
AVN	16 (14.5)	5 (12.2%)	4 (11.4%)	7 (20.6%)
Nonunion	5 (4.5%)	2 (4.9%)	1 (2.9%)	2 (5.9%)
Reoperations^1^	18 (16.4%)	5 (12.2%)	3 (8.6%)	10 (29.4%)

AVN: avascular necrosis of the femoral head. ^**1**^Conversion of internal fixation to total hip replacement.

**Table 4:** Univariate analysis of risk for complications and reoperations.

Variables	Without complications (*n* = 86)	With complications (*n* = 24)	*p*	Without reoperations (*n* = 92)	With reoperations (*n* = 18)	*p*
Age	51.10 ± 11.04	52.58 ± 7.99	1.000^1^	51.03 ± 11.02	53.44 ± 6.59	0.824^1^
Sex			0.101^2^			0.265^2^
Female	34 (30.9%)	14 (12.7%)		38 (34.5%)	10 (9.1%)	
Male	52 (47.3%)	10 (9.1%)		54 (49.1%)	8 (7.3%)	
Side			0.356^2^			0.303^2^
Right	41 (37.3%)	14 (12.7%)		44 (40.0%)	11 (10.0%)	
Left	45 (40.9%)	10 (9.1%)		48 (43.6%)	7 (6.4%)	
Garden classification			0.046^2^			0.067^2^
I, II	24 (21.8%)	2 (1.8%)		25 (22.7%)	1 (0.9%)	
III, IV	62 (56.4%)	22 (20.0%)		67 (60.9%)	17 (15.5%)	
Pauwels classification			0.130^2^			0.122^3^
I	23 (20.9%)	2 (1.8%)		24 (21.8%)	1 (0.9%)	
II	42 (38.2%)	13 (11.8%)		45 (40.9%)	10 (9.1%)	
III	21 (19.1%)	9 (8.2%)		23 (20.9%)	7 (6.4%)	
Fracture level			0.820^3^			1.000^3^
Subcapital	48 (43.6%)	15 (13.6%)		52 (47.3%)	11 (10.0%)	
Midcapital	31 (28.2%)	8 (7.3%)		33 (30.0%)	6 (5.5%)	
Basicervical	7 (6.4%)	1 (0.9%)		7 (6.4%)	1 (0.9%)	
Reduction classification			0.020^2^			0.043^2^
Anatomic reduction	35 (31.8%)	6 (5.5%)		36 (32.7%)	5 (4.5%)	
Positive buttress position reduction	30 (27.3%)	5 (4.5%)		24 (21.8%)	10 (9.1%)	
Negative buttress position reduction	21 (19.1%)	13 (11.8%)		32 (29.1%)	3 (2.7%)	
Garden alignment index angles						
AP	164.5 ± 3.6	163.7 ± 3.7	0.316^1^	164.3 ± 3.7	164.3 ± 3.2	0.796^1^
Lateral	177.7 ± 2.1	176.6 ± 2.9	0.137^1^	177.6 ± 2.1	176.7 ± 3.1	0.523^1^
CTI (every 0.1)			0.026^2^			0.046^2^
0.4-0.5	14 (12.7%)	9 (8.2%)		16 (14.5%)	7 (6.4%)	
0.5-0.6	47 (42.7%)	13 (11.8%)		50 (45.5%)	10 (9.1%)	
0.6-0.7	25 (22.7%)	2 (1.8%)		26 (23.6%)	1 (0.9%)	
TCD			0.954^2^			0.774^2^
<5 mm	64 (58.2%)	18 (16.4%)		69 (62.7%)	13 (11.8%)	
≥5 mm	22 (20.0%)	6 (5.5%)		23 (20.9%)	5 (4.5%)	
Angle of inferior screw			0.005^3^			0.013^3^
<125°	5 (4.5%)	2 (1.8%)		3 (2.7%)	4 (3.6%)	
≥125°	84 (76.4%)	19 (17.3%)		89 (80.9%)	14 (12.7%)	

AP: anterior-posterior; CTI: cortical thickness index; TCD: tip-caput distance. ^1^Mann-Whitney *U*-test; ^2^chi-square test; ^3^Fisher's exact test.

**Table 5:** Multivariate logistic regression analysis of risk for complications.

Variables	Adjusted OR (95% CI)	*p*
Garden classification		
I, II	1	
III, IV	3.226 (0.402 to 25.882)	0.270
Reduction classification		
Anatomic reduction	1	
Positive buttress position reduction	2.003 (0.386 to 10.390)	0.408
Negative buttress position reduction	4.309 (1.137 to 16.322)	0.032
CTI (every 0.1)		
0.4-0.5	1	
0.5-0.6	0.048 (0.005 to 0.510)	0.012
0.6-0.7	0.128 (0.013 to 1.237)	0.076
Angle of inferior screw		
≤125°	1	
>125°	0.043 (0.004 to 0.498)	0.012

CI: confidence interval; CTI: cortical thickness index; OR: odds ratio; TCD: tip-caput distance.

**Table 6:** Multivariate logistic regression analysis of risk for reoperations.

Variables	Adjusted OR (95% CI)	*p*
Garden classification		
I, II	1	
III, IV	6.904 (0.571 to 83.449)	0.129
Reduction classification		
Anatomic reduction	1	
Positive buttress position reduction	1.274 (0.245 to 6.618)	0.773
Negative buttress position reduction	5.744 (1.177 to 28.042)	0.031
CTI (every 0.1)		
0.4-0.5	1	
0.5-0.6	0.033 (0.002 to 0.543)	0.017
0.6-0.7	0.086 (0.006 to 1.298)	0.076
Angle of inferior screw		
≤125°	1	
>125°	0.063 (0.006 to 0.659)	0.021

CI: confidence interval; CTI: cortical thickness index; OR: odds ratio; TCD: tip-caput distance.

## Data Availability

All the data are available in contact with the corresponding author.
